# Supplementary material for: Athletes’ Opinions on Food Provision at European Athletics Championships: Implications for the Future
Source: Nutrients. 2023 Jan 13;15(2):413. doi: 10.3390/nu15020413 (PMC9863385; doi:10.3390/nu15020413)
Supplement: Supplementary file 1 [file nutrients-15-00413-s001.zip › nutrients-2150103-supplementary.pdf]

Date (d/m/y): _____/_____/_______ Code: ☐☐☐☐

Athletes' opinion about the catering service and availability of nutritional and dietary information at European Athletics Championships

PART A – DEMOGRAPHIC INFORMATION

Please mark with an X the answer of your choice or write the answer in the space provided.

1. Name: ____
2. E-mail: ____
3. Sex: ☐ Female ☐ Male
4. Date of birth (d/m/y): _____/_____/___________ 4.1. Age: __________________
5. Country of origin: ________________ 5.1. Country representing: _______________
6. Level of education:

☐ Never attended school ☐ Completed intermediate/middle school

☐ Completed high school ☐ Attended university or other tertiary institution

7. Sporting event (s): ____
8. Hotel where you are staying during competition: _______________________________
9. Have you ever competed in an international competition before? ☐ Yes ☐ No

9.1. **If yes**, have you ever eaten in the dining area before? ☐ Yes ☐ No

9.1.1. **If yes**, in which competition(s)? __________________________________

____

10. Do you follow a dietary regimen prescribed by a registered dietitian/nutritionist?

☐ Yes ☐ No

11. Do you have any specific food restriction? ☐ Yes ☐ No

11.1. **If yes**, choose the option that reflects your specific dietary regimen, and describe in which it consists of:

☐ Allergy/avoidance: ____

☐ Vegetarian: ____

☐ Vegan: ____

☐ Red meat avoiders: ____

☐ Religious beliefs: ____

☐ Other(s). Which? ____

PART B – CATERING SERVICE

1. **Factors influencing food choice.** For each one of the items listed below, **mark the response** that best characterizes your opinion about its relevance for your food choice.

	Not important	Less important	So-so	Important	Very important
Smell	☐	☐	☐	☐	☐
Temperature	☐	☐	☐	☐	☐
Visual appearance	☐	☐	☐	☐	☐
Familiar food	☐	☐	☐	☐	☐
Nutrient composition	☐	☐	☐	☐	☐

Cooking method	☐	☐	☐	☐	☐
Stage of competition	☐	☐	☐	☐	☐
Time of the day	☐	☐	☐	☐	☐
Presence of coach	☐	☐	☐	☐	☐
Presence of team mates	☐	☐	☐	☐	☐
Proximity to the hotel	☐	☐	☐	☐	☐
Proximity to the stadium	☐	☐	☐	☐	☐

2. **Menu quality.** For each one of the items listed below, **mark the response** that best characterizes your opinion about the different aspects related to the quality of the menu.

	Very poor	Poor	Average	Good	Very good
Food safety	☐	☐	☐	☐	☐
Food quality	☐	☐	☐	☐	☐
Freshness	☐	☐	☐	☐	☐
Variety	☐	☐	☐	☐	☐
Portion size	☐	☐	☐	☐	☐
Presentation	☐	☐	☐	☐	☐
Taste	☐	☐	☐	☐	☐
Temperature	☐	☐	☐	☐	☐
Capacity to meet your individual needs	☐	☐	☐	☐	☐

3. **Food accessibility.** For each one of the items presented below, **mark the response** that best characterizes your opinion about the accessibility of food and beverages in general.

	Very low	Low	Average	High	Very high
Food	☐	☐	☐	☐	☐
Beverages	☐	☐	☐	☐	☐

4. **Food availability** (i.e., the amount of food/beverages that is physically present in the cafeteria)**.** For each one of the items presented below, **mark the response** that best characterizes your opinion about the availability of food and beverages in general.

	Very low	Low	Average	High	Very high
Food	☐	☐	☐	☐	☐
Beverages	☐	☐	☐	☐	☐

5. **Availability of different food groups.** For each one of the items presented below, **mark the response** that best characterizes your opinion about the overall **amount of food** provided. If you didn't consume a certain type of food listed, circle the option "unsure/did not eat".

	Not available	Not enough	Enough	More than enough	Unsure/did not eat
Vegan	☐	☐	☐	☐	☐
Vegetarian	☐	☐	☐	☐	☐
Dairy free	☐	☐	☐	☐	☐
Gluten free	☐	☐	☐	☐	☐
Lactose free/low	☐	☐	☐	☐	☐
Fish dishes	☐	☐	☐	☐	☐
Meat dishes	☐	☐	☐	☐	☐

Fresh fruit	☐	☐	☐	☐	☐
Vegetables (raw/cooked)	☐	☐	☐	☐	☐
Sports specific foods	☐	☐	☐	☐	☐
"Grab and go" food	☐	☐	☐	☐	☐
Sweets/Desserts	☐	☐	☐	☐	☐
Cultural restrictions/acceptability: ____________________________	☐	☐	☐	☐	☐
Other dietary regime/intolerance: ____________________________	☐	☐	☐	☐	☐

6. Do you think that the different meal options were well identified? **Mark the response** that best characterizes your opinion.

Never	Occasionally	Sometimes	Often	Always
☐	☐	☐	☐	☐

7. **Food search.** How was your search for the foods/meals that meet your nutritional needs and/or dietary requirements (e.g., considering the information available about the items, items' location in the dining area, …)? **Mark the response** that best characterizes your opinion.

Very difficult	Difficult	So-so	Easy	Very easy
☐	☐	☐	☐	☐

PART C – NUTRITIONAL LABELLING

8. Do you know what is a nutritional label? ☐ **Yes** ☐ **No** <u>(if you answered No, go to the PART D)</u>

9. How often do you use nutritional labels? **Mark the response** that best characterizes your opinion.

Never	Occasionally	Sometimes	Often	Always
☐	☐	☐	☐	☐

10. Why don't you use nutritional labels more often? **Mark as many statements as necessary** to characterize your opinion.

10.1. ☐ I don't understand the information presented on nutritional labels.
10.2. ☐ I don't trust the information presented on nutritional labels.
10.3. ☐ I'm not used to reading nutritional labels.
10.4. ☐ I think that the information presented on nutritional labels is not useful.
10.5. ☐ I think that nutritional labels are confusing.
10.6. ☐ I don't need to read nutritional labels.
10.7. ☐ I don't have time to read nutritional labels.
10.8. ☐ Other reason(s). Which? ____

11. In future championships, would you like to have nutritional information regarding the served meal? ☐ **Yes** ☐ **No**

12. Do you consider that the present of nutritional labels would help you make better meal choices? ☐ **Yes** ☐ **No**

12.1. Explain why? ____

13. **Components of the nutritional label.** For each one of the items presented below, **mark the response** that best characterizes your opinion about its relevance on the nutritional label.

	Not important	Less important	So-so	Important	Very important	Unsure/Do not know
Ingredient list	☐	☐	☐	☐	☐	☐
Allergens	☐	☐	☐	☐	☐	☐
Serving size	☐	☐	☐	☐	☐	☐

Energy	☐	☐	☐	☐	☐	☐
Carbohydrates	☐	☐	☐	☐	☐	☐
Sugar	☐	☐	☐	☐	☐	☐
Protein	☐	☐	☐	☐	☐	☐
Fat	☐	☐	☐	☐	☐	☐
Saturated fat	☐	☐	☐	☐	☐	☐
Sodium/salt	☐	☐	☐	☐	☐	☐
Icon representing a special regimen (e.g., fish, gluten free, vegetarian)	☐	☐	☐	☐	☐	☐
Icon representing an allergen	☐	☐	☐	☐	☐	☐
Icon representing the nutritional content of the dish (e.g., high carb, high fat)	☐	☐	☐	☐	☐	☐

PART D – OPEN-ENDED AND GENERAL QUESTIONS

14. Were you able to find/locate the adequate meals to supply your dietary needs? **Mark the response** that best characterizes your opinion.

**Never**	**Occasionally**	**Sometimes**	**Often**	**Always**
☐	☐	☐	☐	☐

15. What do you think could help you find/locate a certain item?

____

16. What were your favourite meals in the menu?

____

____

17. Leave any request about any food you consider important and was missing from the menu and regarding additional items you would like to see in future competitions. Why?

____

____

18. Did your experience in the dining hall live up to your expectations? **Mark the response** that best characterizes your opinion.

**Much less than expected**	**Less than expected**	**Matched**	**Exceeded expectations**	**Greatly exceed expectations**
☐	☐	☐	☐	☐

19. Rate your overall experience eating at the dining area from 1 to 10, where 1 = very bad and 10 = excellent or satisfaction. **Mark the response** that best characterizes your opinion.

**1**	**2**	**3**	**4**	**5**	**6**	**7**	**8**	**9**	**10**
☐	☐	☐	☐	☐	☐	☐	☐	☐	☐

20. Leave any comments or suggestions for improvement.

____

____

Thank you for taking the time to complete this survey! We appreciate you taking the time to complete this survey.
